# Enhanced Metabolite Productivity of *Escherichia coli* Adapted to Glucose M9 Minimal Medium

**DOI:** 10.3389/fbioe.2018.00166

**Published:** 2018-11-12

**Authors:** Peter Rugbjerg, Adam M. Feist, Morten Otto Alexander Sommer

**Affiliations:** ^1^The Novo Nordisk Foundation Center for Biosustainability, Technical University of Denmark, Kongens Lyngby, Denmark; ^2^Department of Bioengineering, University of California, San Diego, San Diego, CA, United States

**Keywords:** productivity, glycolytic flux, platform strain, adaptive laboratory evolution, mevalonic acid

## Abstract

High productivity of biotechnological strains is important to industrial fermentation processes and can be constrained by precursor availability and substrate uptake rate. Adaptive laboratory evolution (ALE) of *Escherichia coli* MG1655 to glucose minimal M9 medium has been shown to increase strain fitness, mainly through a key mutation in the transcriptional regulator *rpoB*, which increases flux through central carbon metabolism and the glucose uptake rate. We wanted to test the hypothesis that a substrate uptake enhancing *rpoB* mutation can translate to increased productivity in a strain possessing a heterologous metabolite pathway. When engineered for heterologous mevalonate production, we found that *E. coli rpoB* E672K strains displayed 114–167% higher glucose uptake rates and 48–77% higher mevalonate productivities in glucose minimal M9 medium. This improvement in heterologous mevalonate productivity of the *rpoB* E672K strain is likely mediated by the elevated glucose uptake rate of such strains, which favors overflow metabolism toward acetate production and availability of acetyl-CoA as precursor. These results demonstrate the utility of adaptive laboratory evolution (ALE) to generate a platform strain for an increased production rate for a heterologous product.

## Introduction

Metabolic engineering of microbial production strains often benefits from evolutionary optimization strategies when a desired phenotype is selectable (Nielsen and Keasling, 2016). Adaptive laboratory evolution (ALE) has been shown to increase strain tolerance to toxic substrates and products and enable improved production yields and titers (Dragosits and Mattanovich, 2013). However, since biosynthesis of only a few native products is growth-coupled with optimal energy production (Feist et al., 2010), direct ALE to improve production phenotypes is currently limited, and diverse mutation types instead readily disrupt heterologous product genes during long-term cultivation (Rugbjerg et al., 2018a). Recently, synthetic product selection systems have expanded the possibilities for guiding evolution toward a phenotype: by coupling product-responsive biosensors to fluorescence or selection genes, large mutagenized enzyme, or metagenomic libraries can be interrogated for desirable biocatalytic activities (van Sint Fiet et al., 2006; Raman et al., 2014; Mahr et al., 2015; Genee et al., 2016).

The production rate (i.e., productivity) is an important fermentation process parameter together with yield and titer, and in principle, productivity may be limited by the substrate uptake rate. The specific productivity (mmol/L/gDW) further accounts for biomass differences, which also influences productivity.

Many metabolic products, such as terpenoids, are derived of the central carbon metabolism via acetyl-CoA (Martin et al., 2003). In this work, we hypothesize that the central carbon metabolism of a production host strain can be improved by ALE to yield a substrate-adapted strain with a capacity for heterologous metabolite production at higher rate. The common industrial work-horse *Escherichia coli* strains K-12 and B are lab-manipulated natural isolates (Daegelen et al., 2009), and thus not evolutionarily adapted to minimal M9 glucose medium, which is a cheap and industrially attractive fermentation substrate. Evident of this disconnect, repeated ALE experiments with *E. coli* K-12 have shown how single key mutations significantly specialize the core metabolism and considerably enhance growth rate in minimal medium (Barrick et al., 2009; Conrad et al., 2010). Under a strict glucose M9 minimal medium selection pressure of constant exponential growth, such adaptation can recur via point mutation in the beta subunit of RNA polymerase (e.g., RpoB E672K; LaCroix et al., 2015). Regulatory mutations can quickly change global phenotypes (Philippe et al., 2007). *rpoB* E672K is interesting being a key mutation in adaptation to glucose minimal M9 medium (LaCroix et al., 2015). The mutation enables ≈25% higher growth rates and broadly shifts DNA-binding affinity to globally modulate the host transcriptome (Utrilla et al., 2016). This involves downregulation of unneeded cellular processes in this growth environment (e.g., chemotaxis, flagella formation) and up-regulation of growth-coupled processes favoring glucose uptake and transcription rate (LaCroix et al., 2015; Utrilla et al., 2016). Owing to its enhanced central metabolic flux state, the *rpoB* E672K mutant strain takes up glucose at a higher rate relative to its MG1655 parent and proportionally increases the rates of the central carbon metabolism to also secrete acetate at an increased rate (Long et al., 2017). This altered flux state includes higher flux through the acetyl-CoA node in the route to acetate production.

In this brief report, we test whether the elevated glucose uptake rate of the substrate-adapted *rpoB* E672K mutant strain can be translated into an enhanced production rate of the heterologously-produced metabolite mevalonate.

## Materials and methods

### Strains

The three analyzed production host strains were the ancestral *E. coli* MG1655 wildtype and two isolated mutants from ALE experiments in M9 glucose minimal medium in the study of LaCroix et al. (2015) in which they were genetically validated.

*E. coli* MG1655 (ancestral wildtype used to initiate the original ALE experiment)

*E. coli* MG1655 + *rpoB* E672K

*E. coli* MG1655 + *rpoB* E672K, Δ82bp *pyrE-rph*

Mevalonate-producing versions of the strains were generated by standard electroporation to introduce pMVA1. pMVA1 expresses a mevalonate biosynthetic operon (*E. coli atoB, Lactobacillus casei mvaS*, and *mvaE*) constitutively by the J_23100_ promoter and is propagated by a p15A origin of replication and selected for by chloramphenicol (Rugbjerg et al., 2018a).

### Medium

M9 minimal medium was supplemented with 0.8% glucose and 0.4% casamino acids: 12.8 g/L Na_2_HPO_4_ x7H_2_O, 3 g/L KH_2_PO_4_, 0.5 g/L NaCl, 1 g/L NH_4_Cl, 2 mM MgSO_4_, 0.1 mM CaCl_2_, 0.4% (w/v) casamino acids (Teknova), 0.5 mM thiamine hydrochloride (Sigma-Aldrich), 8 g/L D-glucose. Thirty milligrams per liter of chloramphenicol for plasmid maintenance.

### Fermentation and sampling

Overnight pre-cultures of the respective strains were grown to stationary phase from single colonies in 3 mL M9 medium supplemented with 0.8% glucose and 0.4% casamino acids at 37°C, 250 rpm shaking.

Medium for 25 mL main cultures were inoculated by 200x back-dilution in 50 mL aerated tubes, cultured at 37°C and shaken horizontally at 250 rpm. Two hundred microliter samples for measurement of OD600 were analyzed on a BioTek Synergy H1 and subsequently converted to gDW by a standard curve. Samples for subsequent chemical analysis were stored at −20°C.

### Chemical analysis

Fermentation samples were analyzed as previously described (Rugbjerg et al., 2018b). In brief, 300 μL samples were thawed and treated with 23 μL 20% sulfuric acid. Samples were vigorously shaken and then spun down at 13,000 g for 2 min. Supernatant (medium) samples were injected into an Ultimate 3000 HPLC running a 5 mM sulfuric acid mobile phase (0.6 mL/min) on an Aminex HPX-87H ion exclusion column (300 × 7.8 mm, Bio-Rad Laboratories) at 50°C. A refractive index detector was used for detection. A standard curve for mevalonate was generated with mevalonolactone (Sigma-Aldrich) dissolved in M9 medium and treated with same relative volume of 20% sulfuric acid.

### Calculation of production and uptake rates

Specific metabolite uptake and production rates were calculated in the indicated regions as the slope of the best linear fit between metabolite (mM) and biomass (gDW/L) concentration, multiplied by the growth rate (1/h) calculated by the best exponential fit between biomass concentration and time in the same region.

## Results

**Figure 1:**
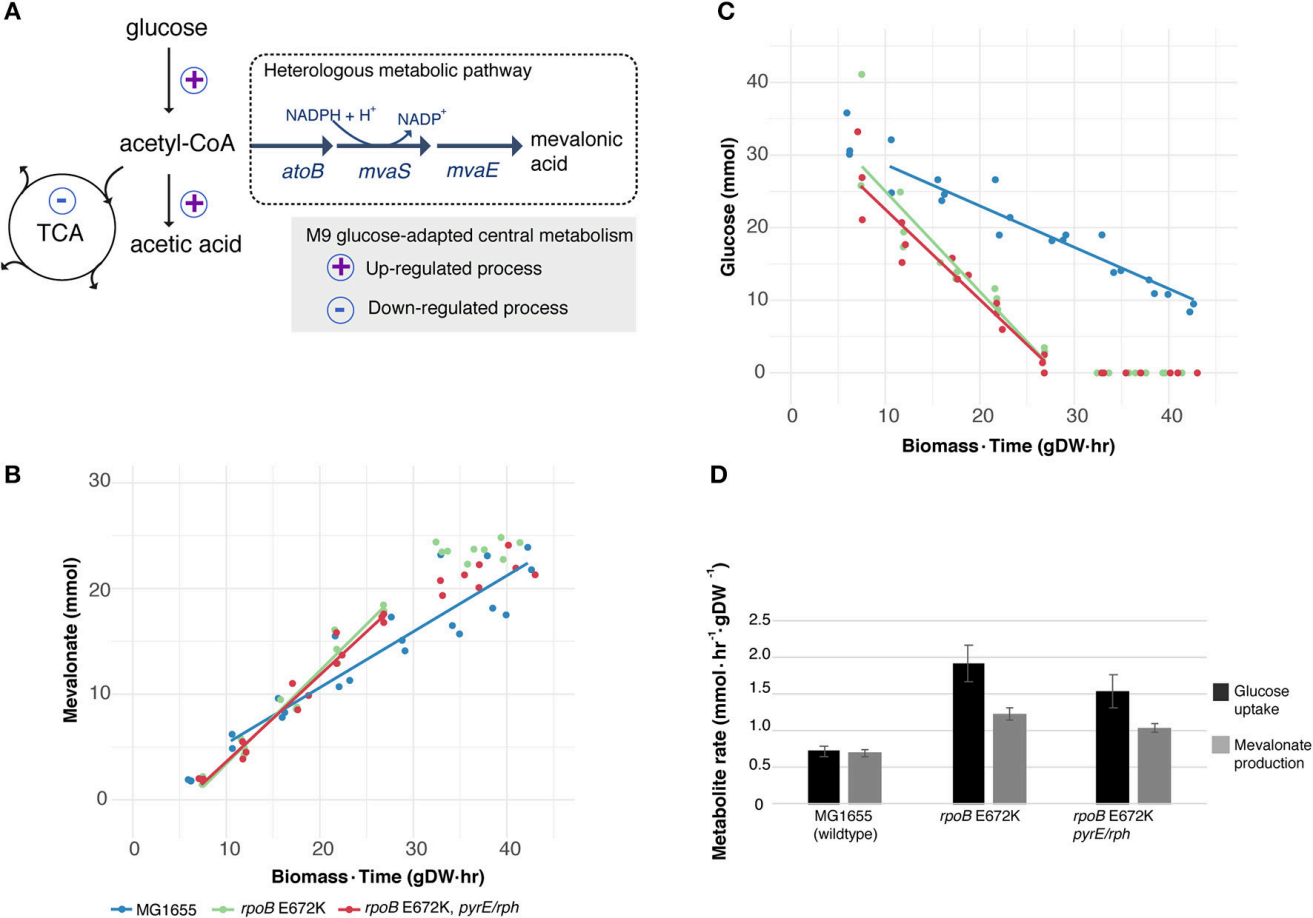
Utilizing a key medium-adapting *E. coli* mutation for heterologous production of mevalonate at higher rate. **(A)** Available substrate flux from acetyl-CoA to mevalonate may be improved in *rpoB*-strains, due to previously observed host adaptation upregulating glucose uptake and fermentative metabolism, while downregulating several cellular functions including the tricarboxylic acid (TCA) cycle (LaCroix et al., 2015). Simplified sketch of central carbon metabolism shown. **(B)** Production of heterologous mevalonate, and **(C)** Glucose consumption in wildtype (MG1655) and glucose M9 minimal medium-adapted (*rpoB*) strains during fermentation. Lines depict average of best linear fits in glucose consumption phase (*n* = 3), points represent replicate measurements. **(D)** Specific uptake and production rate of glucose and mevalonate, respectively, in the glucose to mevalonate conversion phase. Error bars depict standard error of the mean (*n* = 3).

We utilized the improved glucose uptake rates and higher glycolytic flux of medium-adapted *rpoB* E672K mutant strains to test if the higher flux translated to the biosynthesis of heterologous mevalonate, a glycolysis-derived metabolite. Mevalonate is a central biochemical building block for attractive terpenoid products such as fragrances, flavors, and bioplastics (Martin et al., 2003; Xiong et al., 2014). We focus on the *rpoB* E672K mutation, which enables higher growth rates in M9 glucose minimal medium, globally affects the strain proteome and dispenses unneeded functionality (Utrilla et al., 2016). We also included a second adaptive mutation, in *pyrE-rph*, which improves a known pyrimidine biosynthetic defect and additionally increases fitness (Jensen, 1993; LaCroix et al., 2015) to test if its additional gain in fitness (LaCroix et al., 2015) can translate into improved metabolite production. In these two strains and the ancestral MG1655 strain, we therefore introduced a constitutive heterologous metabolic pathway from acetyl-CoA to mevalonate (Figure 1A) based on previously identified enzymes of *Lactobacillus casei*, 3-hydroxy-3-methyl-glutaryl-coenzyme A *(*HMG-CoA) synthase (*mvaS)*, and HMG-CoA reductase (*mvaE*) (Xiong et al., 2014).

Batch growth screens of the medium-adapted strains yielded an improved productivity over the wild type MG1655 starting strain. In M9 minimal medium with 0.8% glucose and 0.4% casamino acid, these two mutant strains exhibited ~10% higher growth rates when engineered with the mevalonate production pathway compared to the ancestor MG1655 carrying the same pathway plasmid (Table 1). Comparing these pathway-engineered mutant and ancestor strains (Table 1) to their pathway-free counterparts, overall lower growth rates of ~35–41% were observed with the pathway plasmid (LaCroix et al., 2015). Production-related decreases in the growth rates are not unexpected given the pathway intermediate toxicities of mevalonate biosynthesis (Kizer et al., 2008) along with the burden of the plasmid in the strains and the subtle differences in batch culturing setups used between the different studies (LaCroix et al., 2015).

**Table 1:** Mean growth rates and specific uptake and production rates of central metabolites and mevalonate, calculated in the glucose to mevalonate conversion phase, ± standard error of the mean (*n* = 3).

**Strain (production phase)**	**Initial growth rate, prior to production (h^−1^)**	**Glucose uptake rate (mmol gDW^−1^ h^−1^)**	**Mevalonate production rate (mmol gDW^−1^ h^−1^)**	**Acetate production rate (mmol gDW^−1^ h^−1^)**	**Lactate production rate (mmol gDW^−1^ h^−1^)**	**Succinate production rate (mmol gDW^−1^ h^−1^)**
MG1655 (10.8–28.4 h)	0.518 ± 0.014	0.7 ± 0.1	0.7 ± 0.0	<0.1	<0.1	<0.1
*rpoB* E672K (7.7–19.8 h)	0.584 ± 0.016	1.9 ± 0.3	1.2 ± 0.1	<0.1	1.0 ± 0.1	0.8 ± 0.0
*rpoB* E672K + *pyrE*-*rph* (7.7–19.8 h)	0.579 ± 0.015	1.5 ± 0.2	1.0 ± 0.1	<0.1	1.2 ± 0.1	1.1 ± 0.0

When exploring the use of the ALE derived strains as production chassis equipped with the mevalonate production pathway, we found that the mutant strains channeled the increased glycolytic flux into an increased rate of mevalonate production, resulting in a 77% increase in specific rate of glucose to mevalonate conversion in *rpoB* E672K relative to MG1655, and a 48% increase in *rpoB* E672K+*pyrE-rph* (Table 1 and Figure 1B). The *rpoB* mutants displayed a 114–167% higher specific glucose uptake rate than MG1655 (Table 1 and Figure 1C). In addition to increased mevalonate production rates with the *rpoB* mutations, the faster initial growth (when production is not detected) may also contribute to faster formation of product in a bioprocess, though the main gain in production speed appears to arise from the improved specific productivity.

Unlike the pathway-free *rpoB* mutants (LaCroix et al., 2015), the mevalonate-producing *rpoB* mutants did not secrete significant amounts of acetate (Table 1). This indicates that these strains diverted the increased flux to acetyl-CoA to mevalonate. However, the increase in mevalonate production rate did not proportionally follow the increase in glucose uptake rate (Figure 1D). Instead, during the mevalonate production phase when glucose was present, the mutants intermittently secreted lactate and succinate as byproducts (Table 1), followed by reuptake upon glucose depletion (Supplementary Figures S1–S3). This intermittent lactate and succinate accumulation is an indication that there is likely a flux limitation or redox imbalance in the engineered pathway to fully utilize the increased glycolytic flux. Future optimization should target this to take full advantage of the gain in productivity without a trade-off in carbon yield. There are many examples of elimination of both lactate and succinate as byproducts which can be examined (King et al., 2017).

Increased productivity appeared to be the main outcome of the implemented *rpoB* E672K mutation while end-point titers following 48 h of cultivation did not significantly differ between the different strains. This also demonstrated no overall difference in carbon yield: all strains accumulated titers of 24–26 mM mevalonate, equivalent of an overall yield of 54–59 mol/mol, which is similar to previous batch fermentation in M9 minimal medium using an inducible pathway with the same enzyme homologs (Xiong et al., 2014). Furthermore, we chose 37°C for this demonstration to match the ALE condition at which the mutant strains were generated, however for optimal mevalonate production, cultivation temperatures around 30°C are usually deployed (Martin et al., 2003; Tabata and Hashimoto, 2004; Xiong et al., 2014). Since the *rpoB* E672K mutant strain maintains a growth advantage over MG1655 at 30°C (data not shown), it is possible that productivity improvements are also seen at this temperature.

## Discussion

Process productivity is an important parameter in the design of economic bioprocesses, as it largely determines the bioreactor volume required (Ikeda, 2003). Thus, an improved productivity can significantly decrease the capital investment necessary for a given process. As shown in this study, improvements in productivity can result from ALE selecting for improved growth rates. The improved specific productivities in the adapted *rpoB* mutant strains examined here may be explained by the global adaptation of the metabolism to minimal M9 medium in excess glucose. The *rpoB* E672K mutation reduces the expression levels of functions involved in environment and stress tolerance, while it increases exponential-phase rates of glucose uptake and acetate production (Utrilla et al., 2016). Overall, this adaptation likely favors an increased availability of acetyl-CoA for the first specific step of the mevalonate product pathway. Addition of the *pyrE-rph* mutation to *rpoB* E672K did not appear to significantly improve productivity further (Figure 1D). This non-increase is likely due to the finding that the *pyrE-rph*-associated fitness gain resulted from restoration of a local biosynthetic defect of MG1655 (Jensen, 1993). Thus, the effect of this mutation appears to be specific to this biosynthetic pathway and does increase growth rate, but not the glucose uptake rate (LaCroix et al., 2015). Improvement of glycolytic flux is generally attractive as a means to increase productivity of a number of metabolic production processes, e.g., ethanol, lactic acid, and several amino acids (Koebmann et al., 2002). In this work, we demonstrate how a single adaptive point-mutation in the global transcriptional regulator RpoB can elevate the specific glucose uptake rate to increase heterologous mevalonate productivity. The results of the strategy are supported by rationally optimized productivities of pyruvic acid and mevalonate, respectively, by increased glucose uptake via specific deletions of the genes *atpFH* (ATP synthase F and H subunits) and *sucA* (2-oxoglutarate dehydrogenase) (Causey et al., 2004; Wang et al., 2016). Recent mevalonate production studies have reached titers of up to 594 mM (88 g/L), yet do not report specific productivities (Xiong et al., 2014). However, high volumetric productivities of, respectively, 6.8 and 13.5 mmol/L/hr were reached in shake flask and 1.3 L fedbatch bioreactors at 30°C, respectively (Xiong et al., 2014; Wang et al., 2016). These volumetric productivities were operating at higher cellular densities which complicates direct comparison.

ALE experimentation for strain design has the potential to sample a larger mutation space than rational engineering as it does not require *a priori* knowledge of what to engineer in a given strain. However, ALE requires a selectable feature. As shown in this brief report, ALE-based substrate and media adaptation is a simple strategy to generate a platform strain with improved productivity of a heterologous metabolite, as resources can be diverted from cellular processes not needed for growth in such a controlled environment to production. Furthermore, recent alternative strategies employing biosensors to screen for improved glycolytic flux (Lehning et al., 2017) and to guide ALE by direct selection for improved production (Mahr et al., 2015) have the potential to make ALE an even more useful engineering tool. However, such approaches are currently limited by the availability of specific biosensors.

The results from this study demonstrate the advantage of using key growth-enhancing mutations found in ALE to optimize host strains toward industrial cultivation medium, which results in an improved production rate of a heterologous metabolite.

## Author contributions

PR, AF, and MS: designed the study; PR: performed experiments; PR, AF, and MS: analyzed the data and wrote the manuscript.

### Conflict of interest statement

The authors declare that the research was conducted in the absence of any commercial or financial relationships that could be construed as a potential conflict of interest.
