# Supplementary material for: Enhanced Metabolite Productivity of Escherichia coli Adapted to Glucose M9 Minimal Medium
Source: Front Bioeng Biotechnol. 2018 Nov 12;6:166. doi: 10.3389/fbioe.2018.00166 (PMC6240765; doi:10.3389/fbioe.2018.00166)
Supplement: Supplementary file 1 [file Data_Sheet_1.PDF]

Supplementary Material

Enhanced metabolite productivity of *Escherichia coli* adapted to glucose M9 minimal medium

Peter Rugbjerg, Adam M. Feist*, Morten Otto Alexander Sommer*

*** Correspondence:** Co-corresponding Authors: msom@bio.dtu.dk

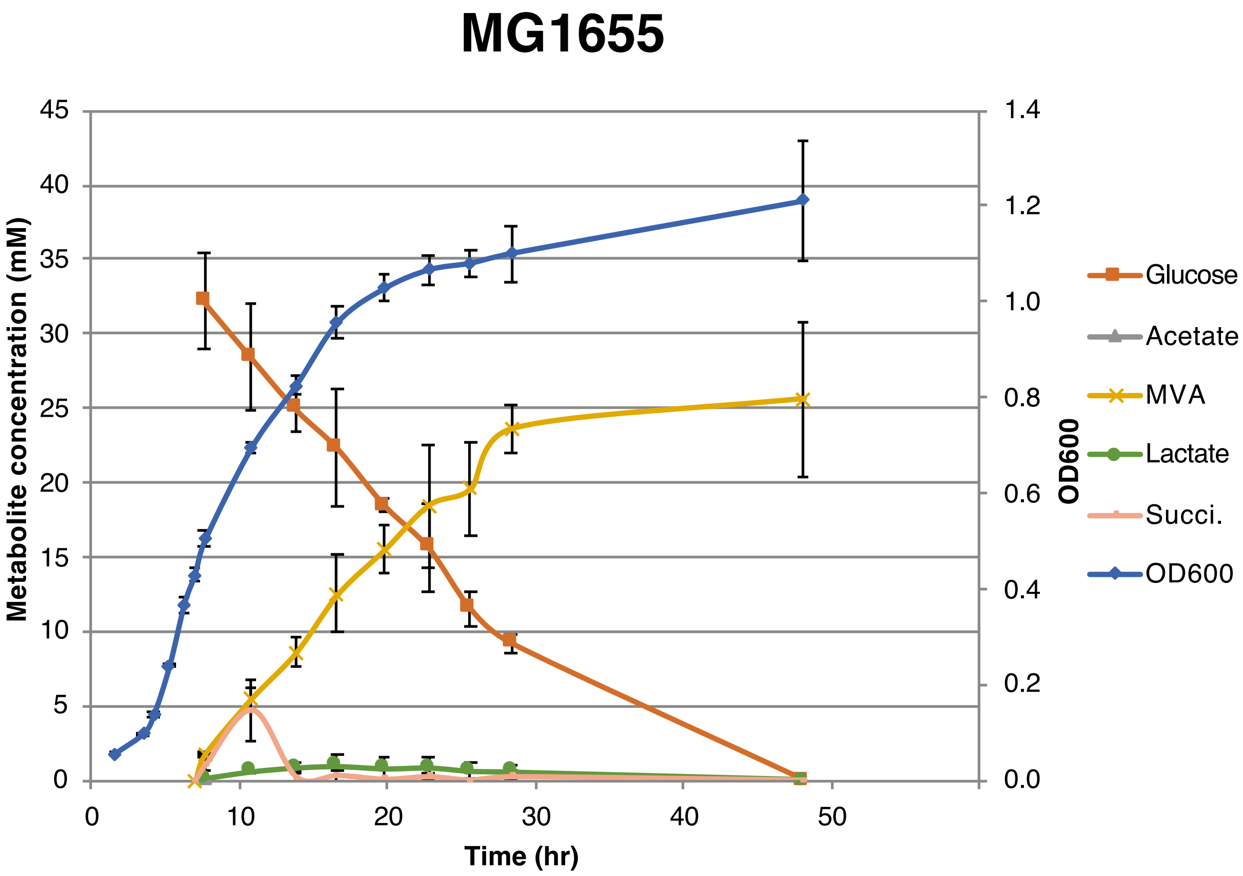

Supplementary Figure S1. Development in cell density (OD600) and metabolite concentration (mM) during fermentation with mevalonate (MVA)-producing MG1655 + pMVA1 in M9 minimal medium with 0.8 % glucose and 0.4 % casamino acids. Error bars indicate standard error (n = 3).

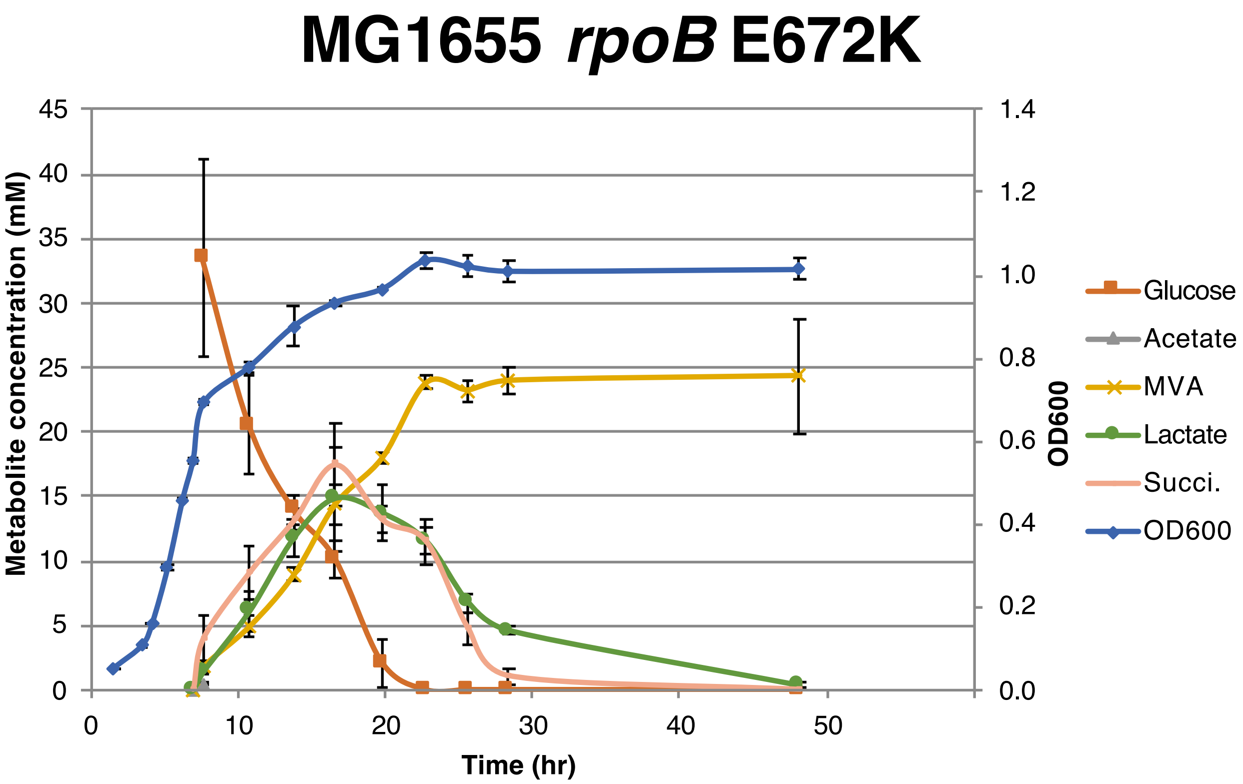

Supplementary Figure S2. Development in cell density (OD600) and metabolite concentration (mM) during fermentation with mevalonate (MVA)-producing MG1655 *rpoB* E672K + pMVA1 in M9 minimal medium with 0.8 % glucose and 0.4 % casamino acids. Error bars indicate standard error (n = 3).

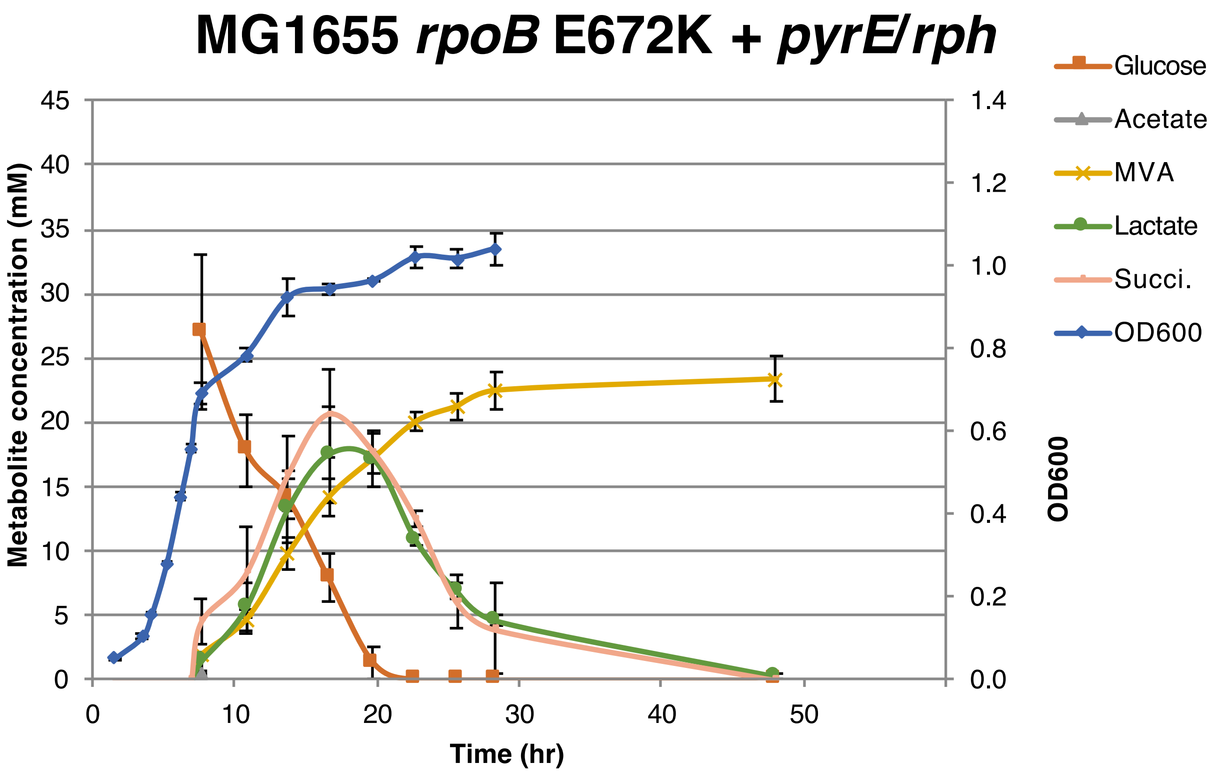

Supplementary Figure S3. Development in cell density (OD600) and metabolite concentration (mM) during fermentation with mevalonate (MVA)-producing MG1655 *rpoB* E672K, *pyrE/rph* + pMVA1 in M9 minimal medium with 0.8 % glucose and 0.4 % casamino acids. Error bars indicate standard error (n = 3).
